# Faith, family, and stigma after parental divorce: psychological well-being among Indonesian Muslim adolescent girls

**DOI:** 10.3389/frcha.2026.1928639

**Published:** 2026-10-02

**Authors:** Slametiningsih, Achir Yani S. Hamid, Mustikasari Mustikasari, Raden Irawati Ismail

**Affiliations:** 1 Doctoral Program in Nursing, Faculty of Nursing, Universitas Indonesia, Depok, Indonesia; 2 Faculty of Nursing, Universitas Indonesia, Depok, Indonesia; 3 Faculty of Medicine, Universitas Indonesia, Jakarta, Indonesia

**Keywords:** adolescent girls, interpretative phenomenological analysis, Islamic cultural context, parental divorce, psychological well-being

## Abstract

**Background:**

Parental divorce can disrupt adolescents’ identity, security, and belonging. In Muslim-majority Indonesia, divorce is also interpreted through cultural expectations of family unity, filial respect, family reputation, and religious meaning. These meanings may intensify stigma, but they may also provide adolescents with moral language for patience, hope, and self-restraint.

**Objective:**

This study explored how Indonesian Muslim adolescent girls make sense of psychological well-being after parental divorce, with attention to Islamic cultural meanings, family relationships, stigma, and coping.

**Methods:**

This qualitative original research used Interpretative Phenomenological Analysis (IPA). Semi-structured face-to-face interviews were conducted with 15 Muslim adolescent girls aged 14–15 years who had experienced parental divorce for at least one year. Participants were recruited purposively from a junior high school in Bekasi City, Indonesia. Interviews were conducted in Bahasa Indonesia, audio-recorded, transcribed verbatim, translated for reporting, and analyzed through idiographic case-by-case interpretation, cross-case theme development, reflexive journaling, and researcher consensus.

**Results:**

Four superordinate themes were identified: negotiating loss and self-acceptance after family rupture; relational ecosystems as both protection and vulnerability; moving from reactive survival toward purposeful autonomy; and Islamic cultural context and moral meaning-making. Participants described divorce as more than a family event. It became a public and moral experience shaped by the label of “broken home,” expectations to respect parents, fear of gossip, and the wish to remain a good Muslim daughter.

**Conclusion:**

In this single-school qualitative study, psychological well-being after parental divorce among Indonesian Muslim adolescent girls was shaped by emotional, relational, cultural, and religious processes. These findings suggest that school and community support should address stigma and emotional distress while engaging respectfully with Islamic family values in context-specific ways.

## Introduction

Parental divorce is not a single moment for adolescents. It can continue through changes in living arrangements, family routines, school events, peer relationships, and a young person's understanding of belonging. Recent longitudinal evidence indicates that adolescents’ internalizing and externalizing problems may increase after parental divorce and persist into later development for some young people (1). Indonesian evidence similarly suggests that parental divorce is associated with emotional difficulties, while qualitative and review studies emphasize the importance of coping, supportive relationships, and resilience (2, 3). However, adolescent responses are not uniform, and adjustment depends on the quality of family relationships, social support, and the meanings adolescents attach to the divorce.

Indonesia provides an important demographic and cultural context for this topic. Official statistics show substantial fluctuations in divorce over the past several years: 291,677 cases were recorded in 2020, increasing to 447,743 in 2021 and 516,344 in 2022, before declining to 463,654 in 2023 and 394,608 in 2024 (4). West Java remains a particularly important setting. BPS recorded 88,842 divorce cases in the province in 2024, while the West Java population-administration profile reported 6,398 divorce records in Kota Bekasi in the same year (5, 6). These figures do not indicate that every adolescent in a divorced family experiences psychological difficulty, but they demonstrate the scale of family transition. For adolescents, divorce occurs within networks of relatives, schools, neighborhoods, and religious communities, so family change may also become a socially visible experience that can be discussed, interpreted, or judged by others.

In Indonesian family life, adolescents’ experiences after divorce occur within a cultural landscape in which family relationships, respect for parents, interdependence, and social harmony are important. Indonesian parenting research describes the influence of traditional cultural values and hierarchical parent–child relationships (7). Recent Indonesian studies likewise describe parental divorce in terms of loss, confusion, emotional distress, self-blame, and resilience, while qualitative research has shown that stigma can extend from divorced parents to their children (2, 8). Thus, divorce may carry meanings beyond the legal end of a marriage. Adolescents may encounter labels such as “broken home,” compare their family with peers’ families, or become concerned about how relatives, classmates, and neighbors interpret their family circumstances.

The Islamic cultural context adds another interpretive layer. In this manuscript, Islamic culture is not treated as a single or homogeneous influence; rather, it refers to religious meanings, family practices, moral expectations, and everyday language that participants may draw upon when interpreting hardship. Religious coping scholarship indicates that faith can provide meaning, hope, and perceived support, while religious coping can also become difficult when adversity is interpreted through guilt, blame, or perceived divine punishment (9, 10). Indonesian qualitative work on adolescents experiencing parental divorce similarly found that acceptance of God's destiny, respect for elders, and family harmony could be incorporated into the process of adjustment (8). In the present study, therefore, concepts such as prayer, sabr, filial respect, and family dignity are treated as participant-described meanings rather than assumed characteristics of all Indonesian Muslim families. This distinction is important because the same value may be experienced as supportive in one interaction and constraining in another.

### Muslim and non-Muslim adolescents: what is currently known

Direct studies comparing Muslim and non-Muslim adolescents’ perspectives on parental divorce are scarce, particularly in Indonesia. Available Indonesian research has generally examined adolescents without religion-based comparison or has focused specifically on Muslim participants. Across these studies, themes such as emotional loss, self-blame, family disruption, and the need for supportive communication appear across adolescents’ experiences (2, 8). The distinctive element in Muslim-focused research is the explicit role of religious meaning: Muslim youth may draw on prayer, acceptance of divine destiny, spiritual comfort, and religious concepts of endurance when interpreting divorce (11, 12). Family expectations concerning respect for parents and family harmony may also be articulated through religious language. However, these observations should not be interpreted as evidence that Muslim adolescents necessarily experience greater stigma or different psychological outcomes than non-Muslim adolescents. Rather, the current evidence suggests that religious coping and faith-based meanings may be more explicitly available within the interpretive repertoire of Muslim participants. A direct Muslim–non-Muslim comparison remains an important area for future research.

Much of the established divorce literature has been produced outside Indonesia, and direct comparative evidence between Muslim and non-Muslim adolescents’ interpretations of parental divorce remains limited. Available Indonesian studies generally examine adolescents across religious backgrounds or focus specifically on Muslim participants rather than directly comparing the two groups. Indonesian qualitative research has emphasized stigma, self-blame, disappointment, and family communication (8), whereas recent phenomenological work among Muslim children has highlighted emotional distress, self-blame, spiritual meaning, and institutional support needs (12). A Malaysian study of Muslim youth has likewise identified Islam as a potential source of spiritual comfort after parental divorce (11). Taken together, these studies suggest that religious meaning may be particularly salient for Muslim adolescents, but they do not justify assuming that Muslim and non-Muslim adolescents respond differently in every respect. Direct comparative studies are still needed. This study therefore focuses specifically on Muslim adolescent girls and examines how faith, family expectations, stigma, and psychological well-being are interpreted together.

Although prior research has examined parental divorce and adolescent adjustment, fewer Indonesian studies have examined how Muslim adolescent girls interpret divorce through psychological, relational, social, and Islamic-cultural meaning systems at the same time. This gap is important because culturally grounded meanings may influence disclosure, help-seeking, coping, and the acceptability of support (13). The present study contributes an idiographic account of girls’ own interpretations rather than a symptom-only description of adjustment. The study aims to: {1} explore how Indonesian Muslim adolescent girls make sense of psychological well-being after parental divorce; {2} examine how family relationships, peer stigma, autonomy, and Islamic cultural meanings shape that experience; and {3} identify context-specific implications for school and family support. The guiding research question was: How do adolescent girls make meaning of parental divorce in relation to self-acceptance, family and peer relationships, autonomy, and Islamic cultural values? The purpose was not to generalize to all Indonesian adolescents, but to provide a culturally grounded account of divorce as lived and interpreted by girls in one urban Indonesian school context.

## Materials and methods

### Study design

This study used Interpretative Phenomenological Analysis (IPA) to examine how participants made sense of parental divorce as a lived experience. IPA was selected because the research question concerned participants’ subjective meaning-making rather than the frequency or distribution of predefined psychological outcomes. Consistent with IPA principles, analysis was idiographic and proceeded case by case before cross-case interpretation. The researchers examined participants’ experiential accounts while also interpreting how participants themselves interpreted their experiences (14–16). The study was reported in line with qualitative research reporting principles and the Consolidated Criteria for Reporting Qualitative Research (COREQ) (17).

### Setting

The study was conducted in a junior high school in Bekasi City, Indonesia. Bekasi is part of the Greater Jakarta metropolitan area and includes families with varied socioeconomic backgrounds, migration histories, and religious practices. The school setting was selected because adolescents spend much of their daily life there and because school is often where family-related stigma, peer comparison, and help-seeking become visible. Interviews were conducted in a private room to protect confidentiality and to reduce the likelihood that participants would feel observed by peers or teachers.

### Participants and sampling

Purposive sampling was used to recruit information-rich participants. Eligible participants were female adolescents aged 14–15 years, identified as Muslim, had experienced parental divorce for at least one year, were enrolled in the participating junior high school, and were willing to talk about their experiences. Adolescents with severe communication difficulties or known acute psychological crises requiring immediate clinical management were not included.

The final sample consisted of 15 adolescent girls. This sample size was considered appropriate for an IPA qualitative study because it provided sufficient information power for the focused research question, the relatively specific participant group, and the depth of interview material (15, 16, 18). Idiographic depth was preserved by reading and interpreting each transcript as an individual case before comparing patterns across cases. The sample size was determined by the focused and relatively homogeneous participant group, the idiographic orientation of IPA, and the depth of the interview material.

### Recruitment and ethical considerations

Initial identification of potentially eligible students was facilitated by a designated school contact. The school contact did not collect interview data and did not have access to transcripts. The research team provided study information to eligible adolescents and their parents or guardians. To reduce coercion, students and parents or guardians were informed that participation was voluntary, that refusal would not affect school relationships or services, and that the school would not receive individual responses. Written parental or guardian consent and adolescent assent were obtained before data collection. Interviews were scheduled in a private room and, where possible, outside core learning activities.

The study was conducted in accordance with the Declaration of Helsinki and received approval from the Health Research Ethics Committee of Sekolah Tinggi Ilmu Keperawatan, approval number No. 88/KEPK-STIKep/III/2026 (19). Because parental divorce is a sensitive topic, the interview protocol included procedures for pausing or stopping interviews if participants became distressed. Participants were informed that they could skip any question, pause the interview, withdraw at any time, and request that their data be removed before de-identification. Referral information for school counseling or appropriate support services was prepared if needed. All names were replaced with participant codes, audio files and transcripts were stored securely, and only the research team had access to de-identified data.

### Data collection

Semi-structured face-to-face interviews were conducted by a trained female researcher with experience in adolescent communication and qualitative interviewing. The interviewer had no teaching or assessment role in the school. Interviews lasted approximately 45–60 min and were conducted in Bahasa Indonesia. The guide began with broad, low-pressure prompts, such as “Can you tell me about your life after your parents separated?” Follow-up questions explored emotional reactions, school experiences, peer relationships, family communication, religious meaning, coping strategies, and future hopes.

The interview format allowed participants to move between difficult memories and present reflections at their own pace. The interviewer used short probes, silence, and reflective listening to support comfort. Interviews were audio-recorded with permission and transcribed verbatim in Bahasa Indonesia. Field notes captured tone, pauses, emotional shifts, and contextual observations. Quotations included in this manuscript were translated into English by a bilingual researcher and reviewed by a second bilingual member of the research team. Translation focused on preserving conceptual meaning and adolescent voice rather than literal word-for-word equivalence. Quotations were lightly edited only to remove fillers, protect confidentiality, and improve readability.

Interview guide prompts included:

- Can you tell me about your life after your parents separated?
- What changed at home, at school, or with friends after the divorce?
- How did you feel when you first understood that your parents would no longer live together?
- How do people around you talk about divorce or “broken home” families?
- Are there religious or family values that influence how you think about your parents’ divorce?
- What helps you cope when you feel sad, angry, confused, or embarrassed?
- What kind of support do you wish adults, teachers, counselors, or family members would give?

### Data analysis

Analysis followed the established stages of IPA (15, 16). First, each transcript was read repeatedly while listening to the corresponding audio recording, with attention to the participant's language, emotional tone, and account of lived experience. Second, exploratory comments were made on descriptive, linguistic, and interpretative features of each transcript. Third, emergent themes were developed from these notes while preserving the idiographic context of each participant. Fourth, connections among emergent themes were examined within each case to form case-level structures. Fifth, the same process was repeated for each participant before examining patterns of convergence and divergence across cases. Finally, superordinate themes were developed through cross-case interpretation while retaining attention to individual cases. This sequence was used to avoid treating participants’ accounts as a decontextualized pool of statements.

Coding was conducted manually using a case-based coding matrix containing significant statements, exploratory comments, emergent themes, case-level themes, and cross-case superordinate themes. Two researchers independently reviewed transcripts and preliminary interpretations, after which differences were discussed until consensus was reached. Reflexive notes documented how researchers’ assumptions about divorce, Islam, gender, family obligation, and resilience could shape interpretation. Negative and contrasting cases were actively examined, including accounts in which faith was experienced as supportive and accounts in which religious or family expectations were experienced as constraining. The final thematic map was developed from the case-level and cross-case analytic structures. Thus, the term IPA in this manuscript refers to an actual idiographic, interpretative analytic process rather than simply to a phenomenological label.

### Trustworthiness

Trustworthiness was strengthened through credibility, transferability, dependability, and confirmability strategies (20, 21). Credibility was supported through repeated transcript reading, researcher triangulation, and member checking of summarized interpretations with three selected participants. Member checking confirmed the overall resonance of the themes and led to minor refinements in the wording of the cultural and religious theme. Transferability was supported by thick description of participants’ sociocultural context and school setting. Dependability was supported through an audit trail documenting coding decisions, theme development, and changes made after peer debriefing. Confirmability was strengthened through reflexive journaling, especially around researchers’ assumptions about divorce, Islam, family obligation, gendered expectations, and adolescent resilience.

## Results

### Participant characteristics

Fifteen Muslim adolescent girls participated in this study. All participants were aged 14–15 years, were enrolled in a junior high school in Bekasi City, Indonesia, and had experienced parental divorce for at least one year before the interview. To reduce the risk of identification in a single-school sample, demographic characteristics are reported in aggregate rather than as individual profiles. Participants lived in varied post-divorce family arrangements, including with mothers, grandmothers, fathers, stepfamilies, or extended family members (Table 1).

**Table 1:** Summary of participant characteristics (*N* = 15).

Characteristic	Category	*n*	%
Age	14 years	8	53.3
	15 years	7	46.7
Living arrangement	Mother/mother's household	8	53.3
	Grandmother	3	20.0
	Father/father's household	2	13.3
	Extended family	2	13.3

Participant characteristics are presented only in aggregate to minimize the possibility of identification in a single-school sample. More detailed combinations of age, living arrangement, and family circumstances were not reported because they could increase deductive disclosure risk.

### Superordinate themes

The analysis identified four superordinate themes. The first captured the internal work of loss, self-blame, and self-acceptance. The second showed how relationships could protect adolescents, expose them, or do both at once. The third described a movement from reactive survival toward more purposeful autonomy. The fourth made the cultural and religious layer explicit: divorce was interpreted through Islamic family ideals, social respectability, filial respect, sabr, prayer, and moral meaning-making. The analytic coding matrix is shown in Table 2, and the thematic map is presented in Figure 1.

**Table 2:** Coding matrix: superordinate themes, subthemes, illustrative quotes, and meanings.

Superordinate theme	Subtheme and code	Illustrative quote/excerpt	Illustrative meaning
Negotiating loss and self-acceptance after family rupture	Temporal grief — waiting for a parent to return	“When my dad left last year, I was waiting for him to come back for months.” (P1)	Divorce remained emotionally active after the household changed; participants continued to live with hope, uncertainty, and delayed acceptance.
	Social comparison — comparing family rituals	“I act like I don't care about school events, but I see who has both parents there.” (P1)	Ordinary school and family events made absence visible and reopened grief.
	Self-blame and magical thinking — suffering to reunite parents	“I stopped playing basketball when Dad left because I didn't deserve to have fun anymore… if I suffer enough, they’re going to see how much this hurts.” (P2)	Participants sometimes interpreted their own pain as a way to make sense of, or even reverse, parental separation.
	Cognitive reframing — understanding without emotional closure	“I have two brains — one that understands and one that still hurts.” (P1)	Some girls could cognitively understand the divorce while still carrying emotional pain.
	School as emotional expression — academic disruption and refuge	“Getting top marks was my way of saying, ‘I am not part of your battle.’” (P4)	Schoolwork became both a site where distress appeared and a space where dignity and control could be rebuilt.
Relational ecosystems as both protection and vulnerability	Adult availability — supportive scaffolding	“When I panic about an essay assignment, he is there… it doesn't matter if your parents are together or not; it won't change what you can achieve.” (P2)	Reliable adults helped stabilize participants when family life felt uncertain.
	Adult availability — feeling replaced after remarriage	“The baby gets all the love right now — as if Mom is trying to start over with a better family.” (P5)	Parental remarriage could intensify emotional invisibility and fear of being displaced.
	Peer relationships — shared experience as protection	“My best friend's parents also divorced… she understands it because she experiences it, too.” (P5)	Peers with similar experiences reduced loneliness and normalized difficult feelings.
	Peer relationships — broken-home teasing and public stigma	“When the seniors found out that my dad left, they said no wonder this is why he didn't want me.” (P3)	School could turn divorce into a public label that deepened shame and social withdrawal.
Moving from reactive survival toward purposeful autonomy	Reactive survival — digital withdrawal as escape	“I’d lock myself in my room scrolling for hours. On the internet, no one knew my family was damaged.” (P3)	Avoidance and withdrawal helped participants reduce exposure to pain, even when it limited support.
	Purposeful distancing — selective engagement and boundaries	“I don't avoid family time completely anymore — just the conversations that feel like picking scabs.” (P4)	Autonomy developed as the ability to choose safer forms of contact rather than total withdrawal.
	Future orientation — planning blocked by uncertainty	“I want to go to university but can't even think about next year when I don't know which parent I’ll be living with.” (P5)	Unstable family arrangements made the future difficult to imagine.
Islamic cultural context and moral meaning-making	Family dignity and moral visibility — protecting family reputation	“They just say ‘be patient’ — as though that solves anything.” (P2)	Calls for patience could be comforting, but they could also silence adolescents’ distress when used without emotional listening.
	Filial respect and restraint — avoiding anger toward parents	“I was angry, but I could not say it openly. He is still my father, and I was taught that children must respect their parents even when they feel hurt.” (P8)	Respect for parents could help maintain moral order, but it also made anger difficult to express.
	Religious coping — prayer, sabr, and hope	*“When I felt sad at night, I prayed and asked Allah to make my heart stronger. I tried to be patient because maybe this was a test for me.”* (P6)	Faith offered a language for endurance, hope, and meaning-making.
	Culturally safe support needs — support without judgment	*“I do not want people to judge my family. I just want someone who understands our religion, listens to me, and does not say that I am a bad child because my parents divorced.”* (P13)	Participants needed support that honored Islamic values while giving permission to speak honestly about pain.

**Figure 1:**
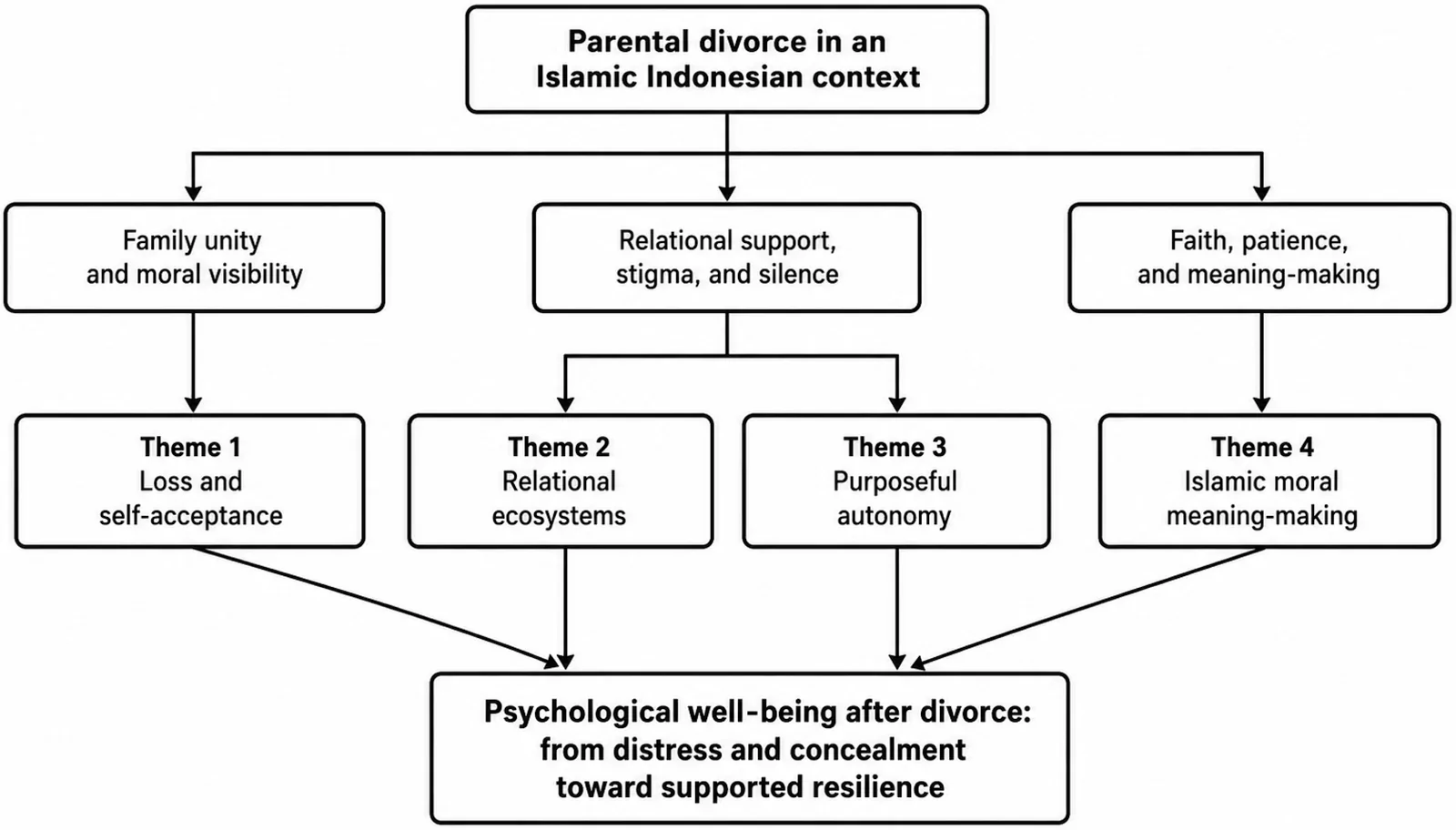
Thematic map for the qualitative study.

### Negotiating loss and self-acceptance after family rupture

Participants did not describe parental divorce as an event that ended once the household changed. Instead, it continued to appear in ordinary moments: birthdays, school ceremonies, quiet evenings, and memories of earlier family routines. For some participants, the absence of a parent remained emotionally active long after the separation. One participant recalled the prolonged hope that her father would return: “*When my dad left last year, I was waiting for him to come back for months”* (P1). Another participant described how school events made the absence of an intact family visible: “*I act like I don't care about school events, but I see who has both parents there”* (P1).

Self-acceptance was not a linear process. Several participants blamed themselves, even when they also understood that the divorce was an adult decision. One participant described punishing herself through withdrawal from activities she once enjoyed: “*I stopped playing basketball when Dad left because I didn't deserve to have fun anymore… if I suffer enough, they’re going to see how much this hurts”* (P2). This statement reflected how distress, guilt, and magical thinking became intertwined in the process of making sense of family rupture.

At the same time, some participants began to develop a more reflective understanding of the divorce, although emotional pain remained. One participant expressed this contradiction clearly: “*I have two brains — one that understands and one that still hurts”* (P1). This excerpt became analytically important because it showed the gap between cognitive understanding and emotional closure.

Academic life carried the same tension. For some participants, school performance declined when grief became overwhelming. For others, achievement became a form of emotional refuge and self-protection. One participant explained: “*Getting top marks was my way of saying, ‘I am not part of your battle’”* (P4). In this sense, schoolwork was not only an educational activity. It became a place where distress appeared, but also a space where dignity, control, and self-worth could be rebuilt.

### Relational ecosystems as both protection and vulnerability

Relationships played a central role in participants’ post-divorce adjustment. They did not simply protect or harm; often, they did both. Supportive adults helped some participants regain a sense of continuity when family life felt unstable. One participant described how academic support from an adult figure helped her feel less defined by her parents’ separation: “*When I panic about an essay assignment, he is there… it doesn't matter if your parents are together or not; it won't change what you can achieve”* (P2).

However, relational support was not consistent across all family contexts. Some participants felt emotionally displaced after parental remarriage or the birth of new siblings. One participant described this feeling of replacement in painful terms: “*The baby gets all the love right now — as if Mom is trying to start over with a better family”* (P5). This form of emotional invisibility made participants question whether they still belonged in the reorganized family.

Peers created another important layer of experience. Friends with similar family backgrounds helped reduce isolation and made divorce feel less like a private defect. One participant stated: “*My best friend's parents also divorced… she understands it because she experiences it, too”* (P5). Shared experience provided emotional validation and helped participants feel less alone.

At the same time, school could also turn divorce into a public label. The phrase “broken home” was not only descriptive; it became a source of stigma. One participant recalled being teased after peers learned about her father's absence: “*When the seniors found out that my dad left, they said no wonder this is why he didn't want me”* (P3). Such experiences contributed to shame, withdrawal, and the desire to hide family problems from others.

### Moving from reactive survival toward purposeful autonomy

Some coping strategies began as immediate survival responses. Participants withdrew, avoided family conversations, spent long periods online, or became quiet when home felt emotionally unsafe. These behaviors were not signs of indifference. They were attempts to reduce exposure to pain. One participant described digital withdrawal as a way to escape the visibility of family rupture: “*I’d lock myself in my room scrolling for hours. On the internet, no one knew my family was damaged”* (P3).

Over time, some participants developed more deliberate forms of autonomy. They did not necessarily withdraw from family life completely, but they learned to identify which conversations felt harmful and which relationships felt safe. One participant explained: “*I don't avoid family time completely anymore — just the conversations that feel like picking scabs”* (P4). This reflected a shift from avoidance as escape toward selective engagement as self-protection.

Future orientation also depended strongly on family stability. Participants with reliable adult support were more able to imagine the next school stage, future work, or a more peaceful family life. In contrast, those living with uncertainty found future planning difficult. One participant described this blocked sense of future: “*I want to go to university but can't even think about next year when I don't know which parent I’ll be living with”* (P5). For this participant, the future was not absent; it was suspended by unresolved family arrangements.

### Islamic cultural context and moral meaning-making

Islamic cultural meanings were woven throughout participants’ accounts. They were not always expressed as formal religious doctrine. More often, they appeared in everyday expectations: respecting parents, avoiding harsh speech, protecting family dignity, being patient, praying, and not embarrassing the family. These expectations shaped how participants interpreted divorce and how freely they felt able to speak about their distress.

Divorce was therefore experienced not only as a private family loss, but also as a socially visible and morally charged event. Participants sensed that family rupture could mark them in the eyes of classmates, neighbors, or relatives. Some felt pressure to remain calm and respectful even when they were hurt. One participant described how repeated advice to be patient could feel emotionally insufficient: “*They just say ‘be patient’ — as though that solves anything”* (P2). This account showed the ambivalence of sabr. Patience could be meaningful, but when offered without listening, it could also silence distress.

Participants also described the burden of maintaining respect toward parents while managing anger, disappointment, and sadness. One participant said: “*I was angry, but I could not say it openly. He is still my father, and I was taught that children must respect their parents even when they feel hurt”* (P8). Faith also functioned as a source of endurance. Another participant explained: “*When I felt sad at night, I prayed and asked Allah to make my heart stronger. I tried to be patient because maybe this was a test for me”* (P6). These accounts show that Islam was not experienced simply as a source of stigma or silence. Religious values could increase restraint, but they could also provide comfort, discipline, and hope.

Participants appeared to need support that did not remove religion from their experience, but understood it with sensitivity. One participant stated: “*I do not want people to judge my family. I just want someone who understands our religion, listens to me, and does not say that I am a bad child because my parents divorced”* (P13). This need for culturally safe support was central to the meaning-making process after parental divorce.

## Discussion

This study explored psychological well-being among Indonesian Muslim adolescent girls living with parental divorce. The findings show that post-divorce adjustment was not only an individual emotional process. It was also relational, cultural, and religious. Participants negotiated loss, rebuilt self-acceptance, searched for safe relationships, and tried to imagine a future while carrying the social meaning of divorce in a Muslim-majority context.

The first theme shows that self-acceptance after divorce was a repeated negotiation rather than a fixed endpoint. Participants moved between grief and understanding, between self-blame and cognitive reframing. This finding is consistent with broader evidence that parental divorce is associated with adolescent emotional distress, depressive symptoms, anxiety, social withdrawal, and later psychological difficulties (1, 22–24). The present study extends this literature by showing how self-blame may persist even when adolescents intellectually understand that the divorce was not their fault.

The second theme highlights the importance of relational ecosystems. Participants were not adjusting alone. Their well-being was shaped by caregivers, stepparents, grandparents, siblings, peers, teachers, and the school environment. Supportive adults helped restore continuity, and peers with similar experiences reduced isolation. However, relational spaces could also become sources of distress when participants felt replaced, neglected, teased, or exposed. These findings align with research showing that post-divorce adjustment depends strongly on relationship quality and social support (25, 26).

### Comparison with Indonesian research and contribution of the present study

The present findings both confirm and extend Indonesian research. Supratman (8), in a qualitative study of Indonesian teenagers aged 18–19 years, identified self-pity, disappointment, and blaming parents after divorce, while interpersonal communication from a single parent and messages concerning acceptance, destiny, respect for elders, and family harmony supported adjustment. Recent mixed-methods research among Indonesian early adolescents similarly identified loss, confusion, anxiety, depression, lower self-esteem, and resilience as important dimensions of post-divorce adjustment (2). A recent phenomenological study of Muslim children in Indonesia further highlighted a broken sense of family, emotional distress, self-blame, and insufficient institutional support (12). The present study extends these findings in three ways. First, it focuses specifically on younger Muslim adolescent girls aged 14–15 years, whose experiences are situated within school life and gendered expectations of being a respectful daughter. Second, it examines stigma, family relationships, autonomy, and Islamic meaning-making within the same idiographic account rather than treating them as separate outcomes. Third, the IPA analysis shows how religious and cultural meanings can operate ambivalently: sabr and prayer may support endurance and hope, while expectations of restraint may also make disclosure difficult. Thus, the contribution is not evidence that Muslim adolescents experience divorce categorically differently from non-Muslim adolescents; rather, it demonstrates how Islamic meanings become part of the lived interpretive context for this specific group.

The third theme suggests that autonomy developed through difficulty. Some participants initially used withdrawal, silence, or avoidance to manage painful family dynamics. Over time, these strategies sometimes became more purposeful, as participants selected safer relationships, set boundaries, and protected emotional space. This finding is relevant for school counselors, pediatric nurses, and adolescent mental health providers. Withdrawal should not automatically be interpreted as indifference. In some cases, it may be an attempt to maintain psychological safety.

The fourth theme is the central cultural contribution of this study. In an Indonesian Islamic cultural context, parental divorce was experienced through ideals of family unity, filial respect, moral reputation, and religious endurance. These findings are consistent with scholarship describing Indonesian family life as shaped by traditional parenting values, interdependence, hierarchy, and respect for parents (7), The stigma of being from a “broken home” made divorce socially visible, echoing earlier Indonesian qualitative evidence that divorce stigma can extend to children (8).

The gendered nature of these experiences also deserves attention. All participants were girls, and their accounts suggested that emotional restraint, family dignity, respectability, and the desire to remain a “good daughter” shaped how they managed distress. The findings should not be interpreted as evidence that girls experience divorce more severely than boys. Rather, they suggest that adolescent girls may face particular expectations to protect family reputation, avoid open anger, and maintain respectful behavior even while experiencing sadness or disappointment. Future studies should compare girls’ and boys’ experiences and examine how gender norms shape silence, disclosure, and help-seeking after parental divorce.

The religious findings should be interpreted as ambivalent rather than one-directional. Cultural and religious values were not simply protective or harmful. When interpreted rigidly, they could intensify silence and make adolescents feel responsible for managing family dignity. When enacted with compassion, they could provide comfort, belonging, and moral strength. This is consistent with religious coping theory, which recognizes that religion can support meaning-making and hope while also contributing to struggle when adversity is interpreted through blame or moral failure (9, 10).

The findings challenge a narrow psychological view of divorce adjustment. Participants were not only managing emotional distress; they were also negotiating reputation, loyalty, school identity, spiritual meaning, and future uncertainty. This is why culturally responsive support matters. By “secular Western” approaches, we refer broadly to counselling models developed primarily within secular clinical traditions, including individually oriented cognitive-behavioral or supportive approaches that may be delivered without explicit religious framing. These approaches should not be assumed to be unsafe or inappropriate for Indonesian Muslim adolescents. Rather, the present findings suggest that some adolescents may experience a counselling encounter as culturally distant when it does not acknowledge filial respect, family dignity, religious language, or the social meaning of divorce. The relevant concern is therefore not that Western-derived models are inherently unsuitable, but that their implementation may require cultural adaptation and collaborative exploration of whether religious meaning is important to the individual adolescent. This interpretation is consistent with Indonesian work describing both general and Islamic counselling approaches and the potential value of integrating psychological and spiritual perspectives in school settings (27, 28).

### Implications for school counselling and culturally responsive support

Because the participants came from one junior high school in Bekasi City, the practical implications should be understood as context-specific rather than as a prescription for all Indonesian schools. In a school with similar characteristics, the findings support several feasible actions. First, school counsellors can provide a confidential pathway for students to disclose family-related distress without using stigmatizing labels such as “broken home.” Second, counsellors and teachers can be alert to subtle changes described in the interviews, including withdrawal, reduced participation, academic disruption, irritability, or achievement used as a way of regaining control. Third, counselling should begin by asking adolescents what meanings they attach to family, religion, and divorce rather than assuming that religious language is either helpful or harmful.

The study does not imply that secular Western-derived counselling models should be replaced by Islamic counselling. Indonesia has multiple counselling traditions in practice. General school guidance may emphasize psychological and academic concerns, while Islamic counselling may explicitly incorporate religious activities, spiritual reflection, and Islamic moral concepts. Research in Indonesian schools has described both approaches and potential integration between them (27). More recent reviews identify approaches such as tazkiyatun nafs, Islamic Cognitive Behavioral Therapy, Islamic expressive writing, and spiritually framed interventions, although evidence quality and implementation capacity vary (28). Indonesian intervention research also illustrates how counselling can integrate Islamic virtues with local cultural norms while retaining structured skills-based techniques, supporting the broader principle of cultural adaptation rather than replacement of psychological methods (29). A recent Indonesian school study also described Islamic guidance and counselling delivered through individual and group activities, while noting challenges such as limited facilities, professional counsellor capacity, and negative perceptions of counselling (30). Therefore, the implication from this study is an integrative option: retain evidence-based psychological counselling skills while adapting communication and, where desired by the adolescent, incorporating culturally and religiously meaningful resources. Such adaptation should remain voluntary, non-coercive, and inclusive. Implications for parents, families, and community support.

The findings also indicate that support should extend beyond the school because adolescents continue to live within post-divorce family arrangements. For parents and caregivers, the first implication is communication. Adolescents may need age-appropriate explanations of divorce, opportunities to ask questions, and reassurance that the separation is not their responsibility. Parent education can emphasize listening before advising and can discourage using adolescents as messengers between parents or asking them to protect adults from emotional consequences.

Religious values such as sabr, obedience, and family dignity may be communicated in ways that support rather than silence emotional expression. For example, patience can be framed as an active capacity to endure difficulty while seeking appropriate support, rather than as an instruction to stop talking about pain. Respect for parents can coexist with permission to express sadness, disappointment, and anger safely. This framing is consistent with the present participants’ accounts, in which religious values were experienced as both meaningful resources and, in some interactions, constraints on disclosure.

Family-based support may also involve parents, grandparents, and other caregivers in identifying stable routines, maintaining predictable contact where appropriate, and protecting adolescents from adult conflict. Community religious leaders may contribute when families want faith-based support, particularly when they reinforce compassion, confidentiality, adolescent welfare, and non-stigmatizing language. However, religious leaders should complement rather than replace professional mental health care when adolescents show persistent distress, functional impairment, or safety concerns. Recent Indonesian work similarly emphasizes collaboration among schools, parents, and communities in Islamic guidance and counselling (30).

### Strengths and limitations

This study has several strengths. It centers the voices of adolescent girls and uses Interpretative Phenomenological Analysis (IPA) to examine how they make meaning of parental divorce. The analysis gives explicit attention to Islamic cultural context, which remains underrepresented in much of the divorce-adjustment literature. The addition of a coding matrix and thematic map strengthens analytic transparency. The study also reports translation procedures and trustworthiness strategies to support the credibility of the findings.

Several limitations should be considered. The sample was homogeneous: all participants were Muslim adolescent girls from one junior high school in Bekasi City. The findings therefore should not be generalized to boys, adolescents from other religious backgrounds, rural settings, or different school systems. The study relied on self-reported experiences, which may be influenced by recall and social desirability. Some quotations were translated from Bahasa Indonesia and lightly edited for readability, which may reduce some of the texture of the original language. The study also did not retain a complete recruitment flow log for all students initially identified by the school contact, limiting the ability to report refusal rates in detail. Finally, because this was a qualitative cross-sectional study, it cannot determine how coping and well-being change over time. Longitudinal and multi-site studies are needed.

## Conclusion

In this single-school qualitative study, Indonesian Muslim adolescent girls described parental divorce as an emotional, relational, cultural, and religious experience. Participants negotiated grief, self-blame, shifting relationships, peer stigma, family dignity, and religious meaning. Islamic values could intensify silence when tied to shame, but they could also support patience, prayer, hope, and moral resilience. The findings suggest that culturally safe school and community support should address stigma, protect confidentiality, and engage respectfully with Islamic values while allowing adolescents to speak honestly about distress.

## Data Availability

The datasets generated and/or analyzed during the current study are not publicly available because they contain sensitive qualitative data from minor participants and public disclosure could compromise participant confidentiality. De-identified data may be made available by the corresponding author upon reasonable request, subject to applicable ethical and institutional requirements.

## References

[1] Tullius JM De Kroon MLA Almansa J Reijneveld SA. Adolescents’ mental health problems increase after parental divorce, not before, and persist until adulthood: a longitudinal TRAILS study. Eur Child Adolesc Psychiatry. (2022) 31(6):969–78. 10.1007/s00787-020-01715-0 33566187 PMC9209384

[2] Dilla YR Wado RD. Understanding the impact of parental divorce on emotional development in early adolescents: a mixed-methods exploration. Acta Psychologia. (2024) 3(2):81–8. 10.35335/psychologia.v3i2.61

[3] Yosep I Mardhiyah A Hikmat R. Adolescent coping strategies with parental divorce: a narrative review. Sci Midwifery. (2022) 10(2):1079–85.

[4] Badan Pusat Statistik. Statistik Indonesia 2025. Jakarta: Badan Pusat Statistik (2025).

[5] Badan Pusat Statistik Provinsi Jawa Barat. Jumlah Perceraian Menurut Kabupaten/Kota dan Faktor Penyebab Perceraian (perkara) di Provinsi Jawa Barat, 2024. Bandung: BPS Provinsi Jawa Barat (2025).

[6] Dinas Kependudukan dan Pencatatan Sipil Provinsi Jawa Barat. Profil Perkembangan Kependudukan Provinsi Jawa Barat Tahun 2024. Bandung: Pemerintah Provinsi Jawa Barat (2025).

[7] Riany YE Meredith P Cuskelly M. Understanding the influence of traditional cultural values on Indonesian parenting. Marriage Fam Rev. (2017) 53(3):207–26. 10.1080/01494929.2016.1157561

[8] Supratman LP. A qualitative study of teenagers’ viewpoint in dealing with parents’ divorce in Indonesia. J Divorce Remarriage. (2020) 61(4):287–99. 10.1080/10502556.2019.1699374

[9] Pargament KI. The Psychology of Religion and Coping: Theory, Research, Practice. New York, NY: Guilford Press (1997).

[10] Abu-Raiya H Pargament KI. Religious coping among diverse religions: commonalities and divergences. Psycholog Relig Spiritual. (2015) 7(1):24–33. 10.1037/a0037652

[11] Orcullo DJC Ismail NAH. The effect of parental divorce on the psychological wellbeing of Muslim youth: implications for counseling and psychotherapy. IIUM J Educ Stud. (2013) 1(1–2):55–97. 10.31436/ijes.v1i1-2.16

[12] Mtd MG. Exploring the lived experiences of psychosocial trauma among Muslim children of divorced parents in Indonesia. Hukmuna J Law Policy. (2025) 1(5).

[13] Rich E Butler-Kruger L Roman N. Exploring strategies to support adolescent mental health after parental divorce: a scoping review. Youth. (2023) 3(1):428–36. 10.3390/youth3010029

[14] Smith JA Osborn M. Interpretative phenomenological analysis as a useful methodology for research on the lived experience of pain. Br J Pain. (2015) 9(1):41–2. 10.1177/2049463714541642 26516556 PMC4616994

[15] Pietkiewicz I Smith JA. A practical guide to using interpretative phenomenological analysis in qualitative research psychology. Psychol J. (2014) 20(1):7–14. 10.14691/CPPJ.20.1.7

[16] Smith JA Flowers P Larkin M. Interpretative Phenomenological Analysis: Theory, Method and Research. 2nd ed London: Sage (2021).

[17] Tong A Sainsbury P Craig J. Consolidated criteria for reporting qualitative research (COREQ): a 32-item checklist for interviews and focus groups. Int J Qual Health Care. (2007) 19(6):349–57. 10.1093/intqhc/mzm042 17872937

[18] Malterud K Siersma VD Guassora AD. Sample size in qualitative interview studies: guided by information power. Qual Health Res. (2016) 26(13):1753–60. 10.1177/1049732315617444 26613970

[19] World Medical Association. World medical association declaration of Helsinki: ethical principles for medical research involving human subjects. JAMA. (2013) 310(20):2191–4. 10.1001/jama.2013.281053 24141714

[20] Lincoln YS Guba EG. Naturalistic Inquiry. Newbury Park, CA: Sage (1985).

[21] Stahl NA King JR. Expanding approaches for research: understanding and using trustworthiness in qualitative research. J Dev Educ. (2020) 44(1):26–8.

[22] Amato PR. Research on divorce: continuing trends and new developments. J Marriage Fam. (2010) 72(3):650–66. 10.1111/j.1741-3737.2010.00723.x

[23] Obeid S Al Karaki G Haddad C Sacre H Soufia M Hallit R. Association between parental divorce and mental health outcomes among Lebanese adolescents: results of a national study. BMC Pediatr. (2021) 21:455. 10.1186/s12887-021-02926-3 34657599 PMC8522189

[24] Sands A Thompson EJ Gaysina D. Long-term influences of parental divorce on offspring affective disorders: a systematic review and meta-analysis. J Affect Disord. (2017) 218:105–14. 10.1016/j.jad.2017.04.015 28463711

[25] Friesen MD Horwood LJ Fergusson DM Woodward LJ. Exposure to parental separation in childhood and later parenting quality as an adult: evidence from a 30-year longitudinal study. J Child Psychol Psychiatry. (2017) 58(1):30–7. 10.1111/jcpp.12632 27481544

[26] Weaver JM Schofield TJ. Mediation and moderation of divorce effects on children’s behavior problems. J Fam Psychol. (2015) 29(1):39–48. 10.1037/fam0000043 25419913 PMC5051343

[27] Gunawan HH Syah M Hasanah A Hadiana ME. Relationship between counseling guidance and Islamic counseling guidance at SMAN 2 Bandung and SMAN 8 Bandung. J Manag Islam Educ. (2023) 1(2):57–68.

[28] Suhertina S. Islamic-based counseling approaches for student mental well-being: a systematic literature review. Madania J Ilmu-Ilmu Keislaman. (2025) 15(2):94–103. 10.24014/jiik.v15i2.37785

[29] Syahril S Janna SR Ratu B Rathnasekara KI. Life skills counseling within local culture-based schools improves Muslim students’ self-acceptance. Pamomong J Islam Educ Couns. (2025) 6(2):155–77. 10.18326/pamomong.v6i2.3785

[30] Fithri MN Nurqadriani N Sumarni S Hamzah MNH. Implementation of Islamic guidance and counseling in addressing adolescent issues in high schools. J Konseling Pendidik Islam. (2026) 7(3). 10.32806/jkpi.v7i3.1265

